# Diacylglycerol kinase γ facilitates the proliferation and migration of neural stem cells in the developing neural tube: DGKγ facilitates NSC migration via the DAG/PKCδ pathway

**DOI:** 10.3724/abbs.2024156

**Published:** 2024-10-23

**Authors:** Huilin Cui, Jiazheng Du, Jianshan Xie, Jixia Zhang, Yun Tao, Yige Huang, Lei Li, Ximei Cao, Yu Zhang

**Affiliations:** 1 Department of Histology and Embryology Shanxi Medical University Taiyuan 030001 China; 2 Key Laboratory of Cellular Physiology Ministry of Education and the Department of Physiology Shanxi Medical University Taiyuan 030001 China

**Keywords:** diacylglycerol kinase γ, protein kinase C, neural tube, neural stem cell, cell proliferation, cell migration

## Abstract

In this study, we aim to investigate diacylglycerol kinase (DGK) γ expression in developing neural tubes (NTs) and its effects on neural stem cell (NSC) proliferation and migration. Whole-mount *in situ* hybridization (WMISH) and immunohistochemistry are performed to explore DGKγ localization in developing NTs *in vivo*. NSCs are treated with sh-DGKγ, R59949, or PMA *in vitro*. Cell counting kit-8 (CCK-8) assay, 5-ethynyl-2′-deoxyuridine (EdU) assay and neurosphere formation assay are utilized to evaluate NSC proliferation. Neurosphere migration assay and a transwell chamber assay are used to assess NSC migration. The diacylglycerol (DAG) content is detected via enzyme-linked immunosorbent assay (ELISA). The mRNA expression of DGKγ is detected via quantitative real-time polymerase chain reaction (qRT-PCR). The protein expression levels of DGKγ, protein kinase C (PKC) and phosphorylated PKC (p-PKC) are detected via western blot analysis. The results show that DGKγ mRNA is expressed predominantly in developing NTs. The neuroepithelium in developing NTs is positive for NSC markers, including Nestin, glial fibrillary acidic protein (GFAP), and DGKγ. DGKγ is expressed in the cytoplasm and nucleus of the neuroepithelium and is coexpressed with p-PKCγ and p-PKCδ. The proliferation of NSCs, the number of EdU-positive NSCs, and the number of neurospheres are decreased by sh-DGKγ and R59949 but increased by PMA. There is a shorter migration distance of NSCs and fewer migrated NSCs in the sh-DGKγ, R59949 and PMA groups. DAG content and the p-PKCδ/PKCδ ratio are increased by sh-DGKγ, R59949 and PMA, whereas the p-PKCγ/PKCγ ratio is decreased by PMA. Taken together, our findings indicate that DGKγ facilitates NSC proliferation and migration, which is responsible for the participation of DGK in NT development. DGKγ facilitates NSC migration via the DAG/PKCδ pathway.

## Introduction

Diacylglycerol kinases (DGKs) terminate diacylglycerol (DAG) signaling via the phosphorylation of DAG to produce phosphatidic acid (PA). To date, ten DGK isozymes have been identified (α, β, γ, δ, ε, ζ, η, θ, ι, and κ), most of which are subtype-specifically expressed in the brain and can regulate brain functions [ 1– 3]. DGKγ is highly expressed throughout the postnatal developmental period [1] and is widely expressed in projection neurons and interneurons of the cerebral cortex, hippocampal formation, and cerebellum [4]; moreover, DGKγ regulates cerebellar long-term depression (LTD) and the dendritic development of Purkinje cells [5]. Our previous study demonstrated that DGKγ is strongly expressed in rapidly developing regions of the rat embryonic brain [6], indicating the importance of DGKγ during brain development. Unfortunately, the physiological roles of DGKγ in different time courses of brain development have not been clearly elucidated. Therefore, the study of DGKγ in the development of NTs is highly important and novel.

The central nervous system (CNS) is the derivative of the neural tube (NT), in which the predominant neuroepithelial cells are neural stem cells (NSCs). The development of NTs and the processes of NSC proliferation and migration are strictly organized and precisely regulated [ 7– 9]. Inositol protects against neural tube defects (NTDs) via the activation of PKC; however, inositol deficiency leads to NTDs [ 10– 12]. As one of the metabolites of inositol, the second messenger, DAG, has numerous targets, the most prominent of which belongs to the protein kinase C (PKC) family [ 13, 14]. Accordingly, it is speculated that the DAG/PKC signaling pathway may regulate NT development.

DGKγ, which has kinase-independent and kinase-dependent functions [5], can function in a kinase-dependent manner by terminating the DAG signaling pathway [15]. Moreover, DGKγ can physically interact with PKCγ and PKCδ [ 16, 17].

In this study, we aimed to explore the expression profile of DGKγ and its potential underlying molecular mechanisms in the development of NTs by using NSCs subjected to *DGKγ* knockdown, R59949 (DGK inhibitor) treatment, or phorbol-12-myristate-13-acetate (PMA, a DAG analog) treatment *in vitro*.

## Materials and Methods

### Animals and embryo collection

Female Sprague-Dawley (SD) rats ( *n*  = 20, 200–250 g) and male SD rats ( *n*  = 5, 300–350 g) aged 8–9 weeks were obtained from the Laboratory Animal Center of Shanxi Medical University (Taiyuan, China). The rats were paired for crossbreeding at 6:00 p.m. The pregnant rat was assumed to be embryonic day 0.5 (E0.5) when a vaginal plug was observed at 6:00 a.m. on the next day, followed by E1 at 6:00 p.m. All procedures were approved by the Animal Ethical Committee of Shanxi Medical University (No. SYDL2022001). Pregnant rats were anesthetized with anhydrous ether, and the embryos from E10 to E11.5 were dissected under a dissection microscope.

### Probe design

On the basis of the mRNA sequence of rat DGKγ in GenBank (accession no. NM_013126.1) and specific cDNA probe sequences of rat *DGKγ* [18], oligonucleotide probes (approximately 40 bp) were designed using Oligo 6 (NBI, Hercules, USA) and checked via BLAST ( https://blast.ncbi.nlm.nih.gov/Blast.cgi). Thereafter, the double digoxygenin (DIG)-labeled antisense RNA probe (5′-TAGCGAGGAAACCTACAGCCTGGAGCCTTACTGGAGTGT-3′) and sense RNA probe (5′-ACACTCCAGTAAGGCTCCAGGCTGTAGGTTTCCTCGCTA-3′) were synthesized by Takara (Dalian, China).

### Whole-mount *in situ* hybridization (WMISH)

WMISH was performed on rat embryos as previously described [ 19, 20]. Briefly, embryos at E10 and E11 were fixed overnight in 4% paraformaldehyde (PFA) at 4°C and then dehydrated with gradient (25%, 50%, 75%, and 100%, 5 min for each) methanol/phosphate-buffered saline tween (PBST) at 4°C. Afterwards, the embryos were rehydrated with a gradient (75%, 50%, 25% and 0%, 5 min for each) of methanol/PBST. Thereafter, the embryos were fixed in 4% PFA containing 0.2% glutaraldehyde for 15 min at 4°C and incubated at 66°C in 1 mL of prehybridization buffer (Boster, Wuhan, China) containing 5 μg of DIG-labeled probe for 24 h. Subsequently, the embryos were incubated with alkaline phosphatase (AP)-conjugated anti-DIG antibody (Roche, Shanghai, China). Color reactions were carried out with NBT/BCIP (Roche). A sense probe served as the negative control (NC). The embryonic structure was determined according to the atlas [ 21, 22]. The samples were photographed under a K-500 stereomicroscope (Motic, Xiamen, China).

### Immunohistochemistry

After being fixed with a mixture of methanol:acetone:water (MAW, 2:2:1, v/v/v), the embryos were dehydrated with gradient ethanol (25%, 50%, 75%, and 100%, 5 min each) at 4°C, cleared with n-butanol, and embedded in paraffin. In brief, transverse sections (7 μm) were mounted on adhesion slides, deparaffinized, and rehydrated with gradient ethanol (75%, 50%, 25% and 0%, 5 min each) at 4°C. Antigens were retrieved in pressure vessels under the following conditions: citrate buffer (pH 7.0), 2 atm, 100°C, 2 min. Afterwards, the sections were treated with 3% H _2_O _2_/methanol for 30 min at room temperature and blocked with 5% bovine serum albumin (BSA) for 1 h at room temperature. The sections were subsequently incubated with primary antibodies against DGKγ (1:1000, sc-49184; Santa Cruz Biotech, Santa Cruz, USA), Nestin (1:1000, sc-377380; Santa Cruz Biotech) and glial fibrillary acidic protein (GFAP, 1:1000, sc-166458; Santa Cruz Biotech) overnight at 4°C, whereas for the control (ctrl), the primary antibodies were replaced by normal goat IgG (1:200, BA1044; Boster) or mouse IgG (1:200, BA1046; Boster). The next day, the sections were incubated with biotinylated rabbit-anti-goat IgG (1:200, BA1006; Boster) or goat-anti-mouse IgG (1:200, BA1001; Boster) for 30 min at room temperature. Immunostaining was visualized by incubation with a streptavidin-biotin-peroxidase complex (Boster) and diaminobenzi-dine (DAB)/H _2_O _2_ (Sigma, Shanghai, China). Images were captured under a light/fluorescence microscope (Olympus, Tokyo, Japan).

### Immunofluorescence staining

The sections were incubated overnight at 4°C with primary antibodies against Nestin (1:1000, sc-377380; Santa Cruz Biotech), DGKγ (1:1000, sc-49184; Santa Cruz Biotech), p-PKCδ (1:500, sc-365969; Santa Cruz Biotech), and p-PKCγ (1:100, 44-975G; Invitrogen, Carlsbad, USA). Thereafter, the sections were incubated with DyLight488-conjugated donkey anti-mouse IgG (1:200, BA1145; Boster), fluorescein isothiocyanate (FITC)-conjugated donkey anti-rabbit IgG (1:300, bs-0295D-FITC; Bioss, Beijing, China), Cy3-conjugated donkey anti-rabbit IgG (1:500, bs-0295D-Cy3; Bioss), and Cy3-conjugated donkey anti-goat IgG (1:500, P0173, Beyotime, Shanghai, China) for 1 h at room temperature and were counterstained with 4′,6-diamidino-2′-phenylindole (DAPI; Sigma) for 10 min at room temperature in the dark. Finally, the fluorescent signals were visualized under a light/fluorescence microscope (Olympus).

### Primary NSC culture

Embryonic NSCs were harvested from the NT tissues of E11 SD rat pups and propagated via the neurosphere method as previously described [23]. NSCs (2 × 10 ^5^ cells/mL) were cultured in 25-cm ^2^ culture flasks containing Dulbecco’s modified Eagle’s medium/nutrient mixture F-12 (DMEM/F12, Gibco, Carlsbad, USA), 2% B27 supplement, 20 ng/mL basic fibroblast growth factor (bFGF), 20 ng/mL epidermal growth factor (EGF) (all from PeproTech, Rocky Hill, USA), 1% penicillin/streptomycin, and 5 μg/mL heparin (Sigma) in an incubator at 37°C with 5% CO _2_. The neurospheres were ready to pass down when the diameter of the majority of the neurospheres reached 100–150 μm. Briefly, neurospheres were dissociated by accutase and gently triturated to obtain single-cell suspensions. NSCs at the third passage were used for the experiments.

### Identification of neurospheres and NSCs *in vitro*

Single-cell suspensions of neurospheres and NSCs were seeded on poly-D-lysine (PDL)-precoated coverslips, cultured for 18 h in an incubator with 5% CO _2_ at 37°C, and then fixed with 4% PFA for 10 min. After being blocked with 3% BSA for 1 h at room temperature, the neurospheres and NSCs were incubated with primary antibodies against Nestin (1:1000, sc-377380, Santa Cruz Biotech) overnight at 4°C and then with FITC-labeled goat anti-mouse IgG (1:100, BA1101, Boster) for 1 h at room temperature. Thereafter, the neurospheres and NSCs were counterstained with DAPI (Sigma) for 15 min at room temperature in the dark. Images were visualized under a light/fluorescence microscope (Olympus).

### NSC grouping

Single-cell suspensions of NSCs (1 × 10 ^5^ cells/mL) were seeded into 6-well plates. Three DGKγ short hairpin (sh)RNAs and one scrambled shRNA were designed with BLOCK-iT™ RNAi Designer ( https://rnaidesigner.thermofisher.com/rnaiexpress/design.do) and produced by Beijing Liuhe Huada Gene Technology (Beijing, China). shRNA was inserted into the pCDH-CMV-MCS-EF1-copGFP lentiviral expression vector (CD511B-1, System Biosciences, Seattle, USA) via a NovoRec® Plus One Step PCR Cloning Kit (NR005, Novoprotein, Shanghai, China). NSCs were infected with scrambled shRNA (sh-NC group) or DGKγ-shRNA (sh-DGKγ group) using Lipofectamine 2000 (Invitrogen), while NSCs that were not infected with lentivirus composed the ctrl group. At 72 h after infection, the NSCs were collected for evaluation of infection efficacy. The scrambled shRNA sequence was as follows: 5′-CTGATGCAGAGCAACACTTCATTCAAGAGATGAAGTGTTGCTCTGCATCAGTTTT-3′. The selected DGKγ-shRNA sequence was as follows: 5′-CGCATTGACAAGGCCAACTTCATTCAAGAGATGAAGTTGGCCTTGTCAATGCTTTT-3′.

Moreover, NSC single-cell suspensions were incubated with vehicle (DMSO; vehicle), 10 μM R59949 (an inhibitor of DGK; R59949; Sigma), or 100 μM PMA (DAG analog to induce PKC activation; PMA; Sigma). After incubation for 18 h at 37°C in an incubator with 5% CO _2,_ the NSCs were collected for subsequent experiments.

### Quantitative real-time polymerase chain reaction (qRT-PCR)

NSC single-cell suspensions were seeded on PDL precoated coverslips and cultured for 18 h in an incubator with 5% CO _2_ at 37°C. Total RNA was extracted from NSCs with TRIzol (Invitrogen). First-strand cDNA was synthesized via the PrimeScript II 1st Strand cDNA Synthesis Kit (Takara, Dalian, China). Quantitative polymerase chain reaction (PCR) was conducted via SYBR Premix Ex Taq II (Tli RNaseH Plus; Takara) on a StepOnePlus Real-Time PCR System (ABI, Foster City, USA). The thermocycling conditions were as follows: predenaturation at 95°C for 30 s, followed by 40 cycles at 95°C for 5 s and 60°C for 10 s. The relative *DGKγ* mRNA level was normalized to that of *β-actin* and calculated via the 2 ^‒ΔΔCT^ method. The sequences of primers used are listed in Table 1.

**Table:** **Table 1** Primer sequences for qRT-PCR

Gene	Primer sequence (5′→3′)	Length
*DGKγ*	F: GTGGGATCCCACAGAGCTCAG R: GACGGAGGAGTTCCCTTCCAC	394 bp
*β-actin*	F: CCCGCGAGTACAACCTTCTT R: CCATCACACCC TGGTGCCTA	195 bp

### Western blot analysis

NSC single-cell suspensions were seeded on PDL precoated coverslips and cultured for 18 h in an incubator with 5% CO _2_ at 37°C. Total protein was extracted from NSCs on ice via radioimmunoprecipitation assay (RIPA) buffer (Boster) supplemented with a phosphatase inhibitor cocktail (Boster). The protein concentrations of the lysates were assessed with a bicinchoninic acid (BCA) protein assay kit (Thermo Fisher Scientific, Waltham, USA). Protein samples were subjected to 8% and 10% SDS-PAGE, followed by transfer to polyvinylidene fluoride (PVDF) membranes (Bio-Rad Laboratories, Hercules, USA). Afterwards, the PVDF membranes were blocked with 5% BSA for 1 h at room temperature. The PVDF membranes were subsequently incubated with primary antibodies against DGKγ (1:500, ab89037; Abcam, Cambridge, UK), p-PKCδ (1:1000, sc-365969; Santa Cruz Biotech), PKCδ (1:1000, sc-213; Santa Cruz Biotech), p-PKCγ (1:1000, 44-975G; Invitrogen), PKCγ (1:1000, BM5623; Boster), and β-actin (1:4000, AP0060; Bioworld Technology, Shanghai, China) overnight at 4°C. The next day, the PVDF membranes were incubated with HRP-labeled goat-anti-mouse IgG (1:5000, BA1051; Boster) or HRP-conjugated goat anti-rabbit IgG (1:5000, BA1055; Boster) secondary antibodies for 1 h at room temperature. Thereafter, the protein bands were developed with an enhanced chemiluminescence (ECL) western blotting substrate (Pierce, Rockford, USA). Band intensities were analyzed via ImageJ (version 1.45; NIH, Bethesda, USA). β-Actin was used as an internal control.

### Cell counting kit 8 (CCK-8) assay

Single-cell suspensions of NSCs (1 × 10 ^4^ cells/well) were seeded in 96-well plates and grown for 24 h in an incubator at 37°C with 5% CO _2_. Then, 10% CCK-8 solution (Beyotime) was added, and the mixture was incubated for another 2 h. Thereafter, the absorbance was recorded at 450 nm with a microplate reader (Bio-Rad Laboratories).

### 5-Ethynyl-2′-deoxyuridine (EdU) assay

NSC single-cell suspensions (1 × 10 ^4^ cells/well) were seeded in 6-well plates coated with 0.1 mg/mL PDL and incubated at 37°C in an incubator with 5% CO _2_. After 48 h, NSCs were incubated with EdU reagent for 2 h and then subjected to EdU incorporation and immunostaining with an EdU staining kit (Meilunbio, Shanghai, China). NSC nuclei were stained with Hoechst 33342 (MA0126; Meilunbio) for 30 min at room temperature. Images were captured under an inverted light/fluorescence microscope (Olympus). The percentage of EdU-positive NSCs was defined as the proliferation rate.

### Neurosphere formation assay

Neurosphere formation was evaluated as described previously [24]. Briefly, single-cell suspensions of NSCs (1 × 10 ^4^ cells/well) were seeded in 6-well plates and incubated for 72 h at 37°C in an incubator with 5% CO _2_. Images were captured under an inverted light/fluorescence microscope (Olympus). The number and size of neurospheres were measured via ImageJ (version 1.45; NIH).

### Neurosphere migration assay

Neurospheres were plated in 6-well plates precoated with 0.1 mg/mL PDL. The neurospheres were subsequently incubated in culture medium with or without PMA/R59949/DMSO for 18 h at 37°C in an incubator with 5% CO _2_. Afterwards, phase-contrast images were captured. As described previously [25], mean migrated distances were measured in 4 directions at a right angle, from the edge of the neurosphere core to the furthest NSCs that migrated out of the neurospheres. Distances were measured blindly by an unbiased observer via ImageJ.

### Single-cell migration assay

Single-cell migration was determined using 24-well transwell chambers with 8-μm pores (Costar-Corning, Corning, USA). The upper chamber was seeded with NSC single-cell suspensions (1 × 10 ^4^ cells/100 μL of DMEM/F12), while the lower chamber was filled with DMEM/F12 containing 20% FBS. After incubation for 6 h at 37°C in an incubator with 5% CO _2_, the migrated NSCs were fixed with 4% PFA for 15 min at 4°C and stained with DAPI (Sigma) for 15 min at room temperature in the dark. Images were captured randomly, and migrated NSCs were counted blindly by an unbiased observer with ImageJ.

### Enzyme-linked immunosorbent assay (ELISA)

NSC single-cell suspensions (1 × 10 ^5^ cells/well) were plated in 6-well plates and incubated for 18 h at 37°C in an incubator with 5% CO _2_. Then, the NSCs were harvested to measure the total DAG content using an ELISA kit (USCN Life Science, Beijing, China) in accordance with the manufacturer’s protocol. A standard curve was generated for each assay.

### Statistical analysis

Experiments were independently repeated at least three times. Data are presented as the mean ± standard deviation (SD). Statistical significance in different groups was analyzed by one-way ANOVA followed by Duncan’s multiple range tests using SPSS (IBM, Armonk, USA). *P*  < 0.05 was considered statistically significant.

## Results

### Spatiotemporal expression of DGKγ in the developing NTs of rats

**Figure 1:**
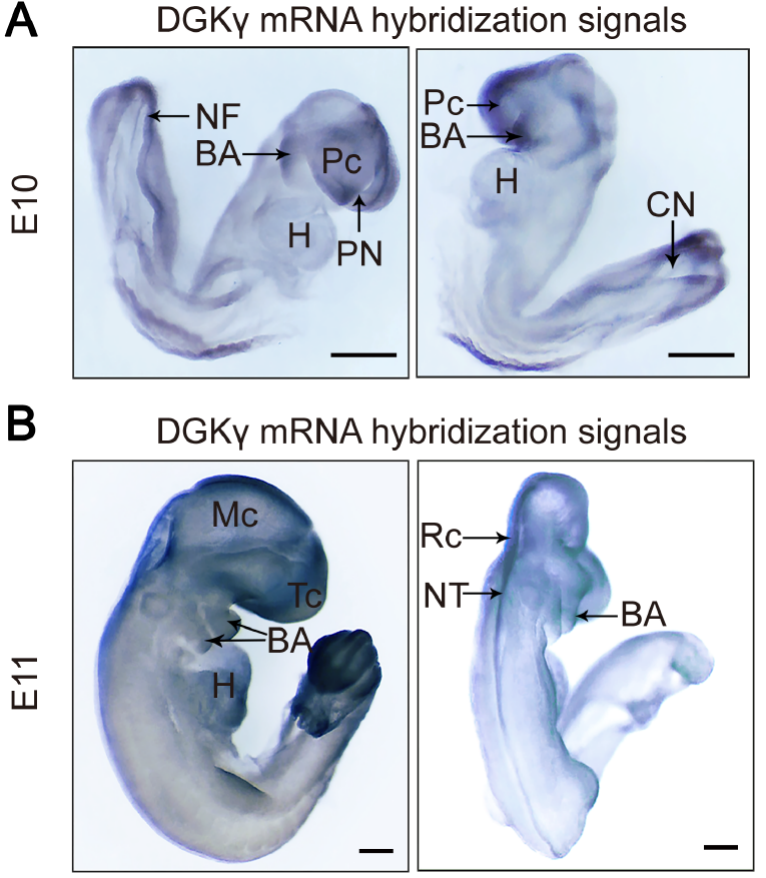
Figure 1 Expression pattern of DGKγ mRNA in rat embryos stained with WMISH The positive signal of WMISH is blue. (A) At E10, DGKγ mRNA hybridization signals were primarily concentrated on the Pc, caudal NF, and BA. (B) At E11, DGKγ mRNA hybridization signals were distributed at the Tc, Mc, Rc, caudal end, H, and BA. BA: branchial arch, CN: caudal neuropore, H: heart, Mc: mesencephalon, NF: neural fold, NT: neural tube, Pc: prosencephalon, PN: prosencephalic neuropore, Rc: rhombencephalon, Tc: telencephalon. Scale bar: 100 μm. n = 5.

During the development of NTs, the formation of NTs was observed at E10 and E11. At E10 ( Figure 1A), DGKγ mRNA hybridization signals were primarily concentrated in the prosencephalon (Pc), branchial arches (BAs), and caudal neural fold (NF). At E11 ( Figure 1B), DGKγ mRNA hybridization signals were predominantly distributed along the NT, especially in the telencephalon (Tc), mesencephalon (Mc), rhombencephalon (Rc), and caudal ends. In addition, the heart (H) and BA were positively stained.

**Figure 2:**
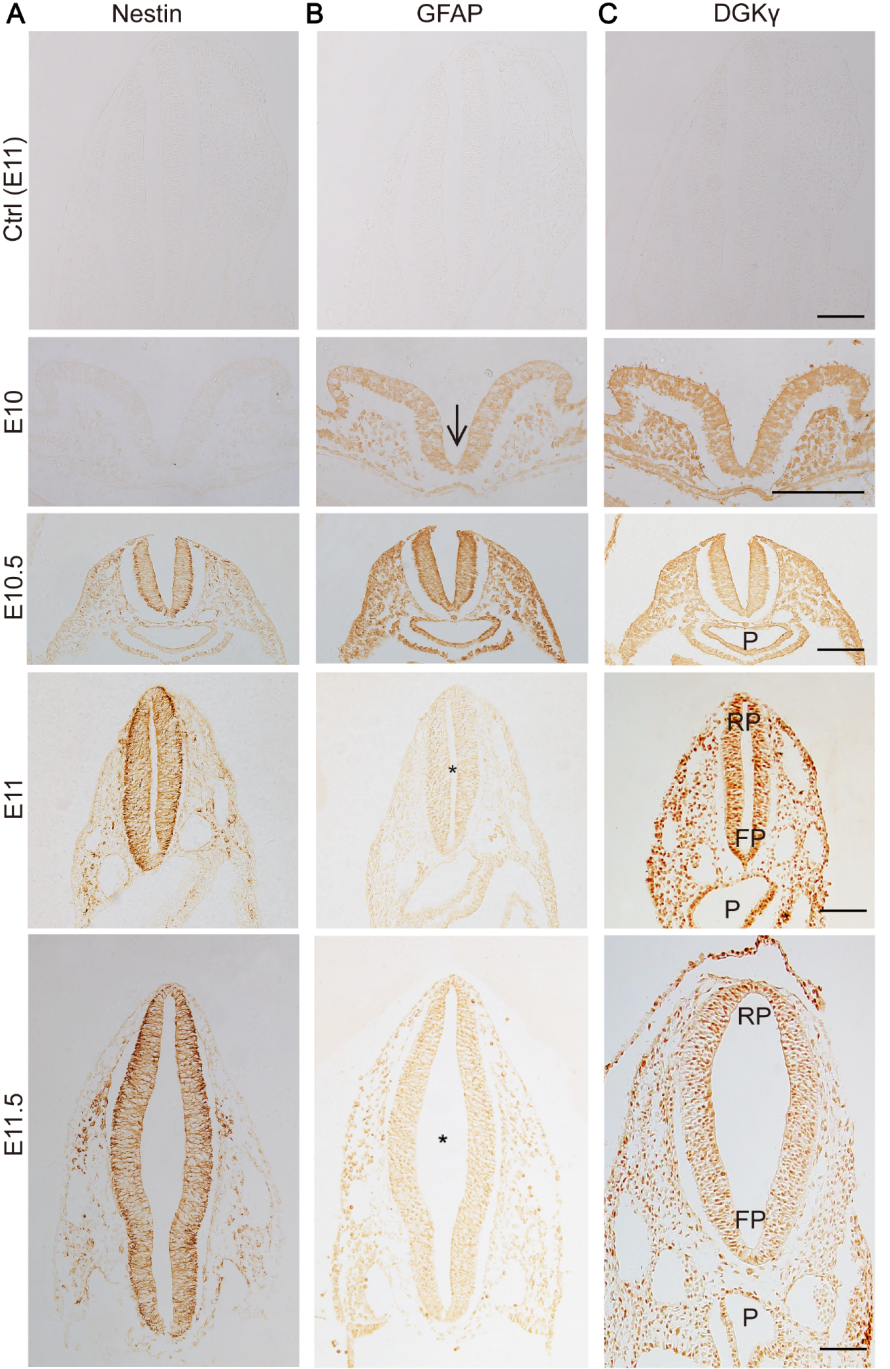
Figure 2 Immunohistochemical staining of Nestin, GFAP and DGKγ in the developing NTs of rat embryos from E10 to E11.5 (A) Nestin emerged in neuroepithelial cells at E10.5 and was consistently expressed from E11 to E11.5. (B) GFAP emerged in neuroepithelial cells at E10, increased gradually from E10 to E10.5, and then decreased at E11 and E11.5. (C) DGKγ was widely expressed in rat embryos at E10 and was consistently expressed from E10 to E11.5. Ctrl: control images were obtained at E11, and the primary antibodies against Nestin, GFAP, and DGKγ were replaced by normal goat IgG or mouse IgG. FP: floor plate, P: primitive pharynx, RP: roof plate, arrows: neural groove, asterisks, NT. Scale bar: 100 μm. n = 6.

Subsequently, immunohistochemistry of transverse sections at the thoracic spine level was used to detect the protein expressions of Nestin, GFAP, and DGKγ during the formation of NTs in rat embryos. Ctrl images at E11 confirmed the specificity of the antibodies, including those against Nestin, GFAP, and DGKγ, which indicated the reliability of the immunohistochemical data. Nestin, a general NSC marker, emerged in the neuroepithelium of the neural groove at E10.5 and was consistently expressed from E11 to E11.5 ( Figure 2A). GFAP, a secondary NSC marker, was expressed in the neuroepithelium at E10 and increased gradually from E10 to E10.5 but decreased at E11 and E11.5 ( Figure 2B). DGKγ immunoreactivity intensity appeared at E10 and increased at E10.5, becoming stronger in the roof plate (RP), upper part and floor plate (FP) at E11, whereas it was concentrated near the RP and became weaker at E11.5. Moreover, DGKγ was widely expressed in rat embryos, including the mesenchyme and endoderm, such as the primitive pharynx (P), and was consistently expressed from E10 to E11.5 ( Figure 2C).

**Figure 3:**
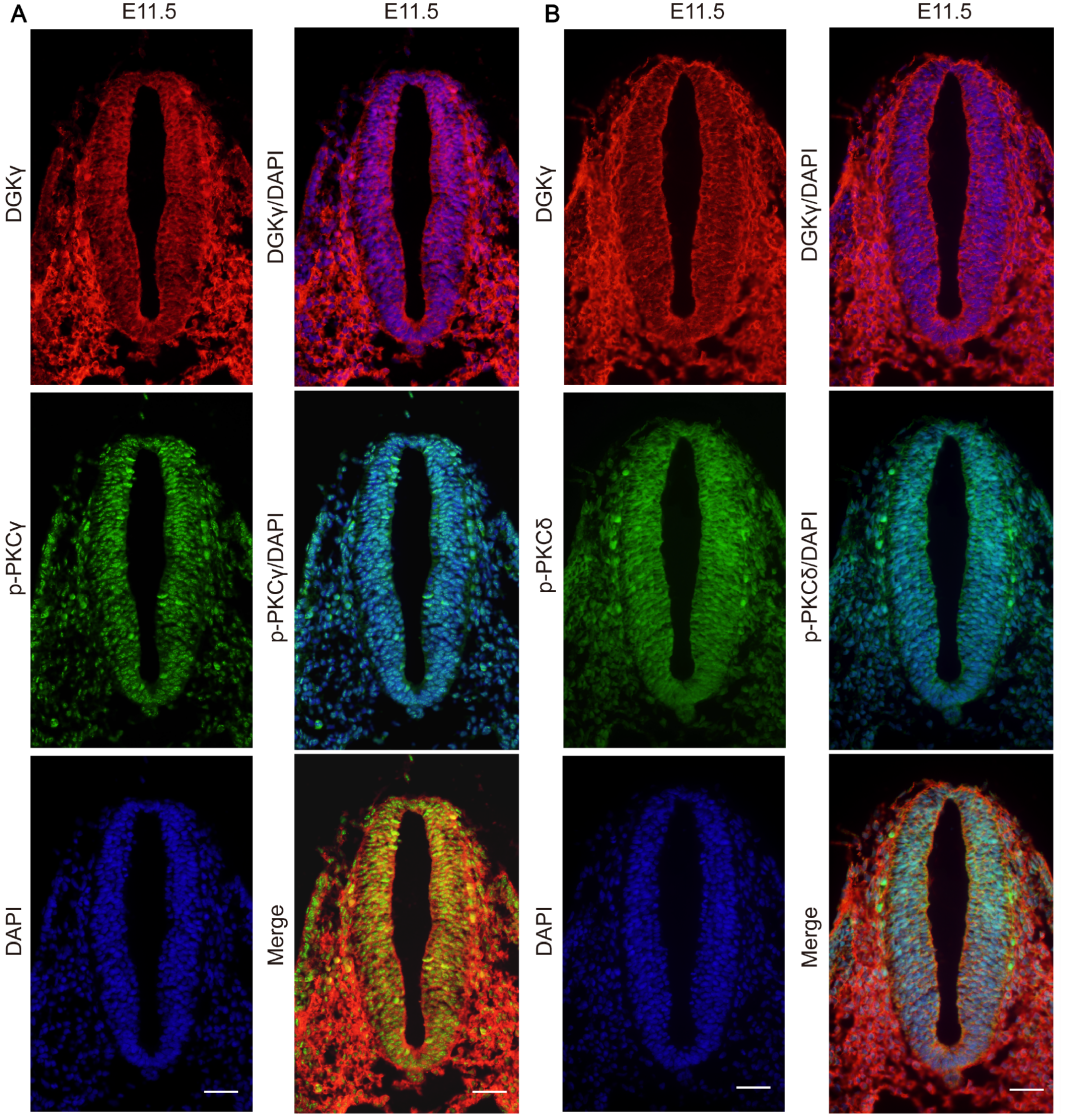
Figure 3 Immunofluorescence staining showing the coexpression of DGKγ with p-PKCγ or p-PKCδ DGKγ was coexpressed with p-PKCγ (A) and p-PKCδ (B) in the cytoplasm and nucleus of neuroepithelial NT cells at E11.5. Blue: DAPI; red: DGKγ; green: p-PKCγ or p-PKCδ. Scale bar: 100 μm. n = 5.

Immunofluorescence staining revealed that DGKγ was expressed in the cytoplasm and nucleus of neuroepithelial cells. Both p-PKCγ and p-PKCδ were detected in the cytoplasm and nucleus of neuroepithelial cells, especially in the nucleus. Collectively, these findings demonstrated that DGKγ was coexpressed with p-PKCγ and p-PKCδ in the cytoplasm and nucleus of neuroepithelial cells ( Figure 3A,B).

These data indicated the spatiotemporal expression of DGKγ in the developing NTs of rats, and DGKγ was coexpressed with p-PKCγ and p-PKCδ in the cytoplasm and nucleus of neuroepithelial cells.

### Identification of neurospheres and NSCs *in vitro*

**Figure 4:**
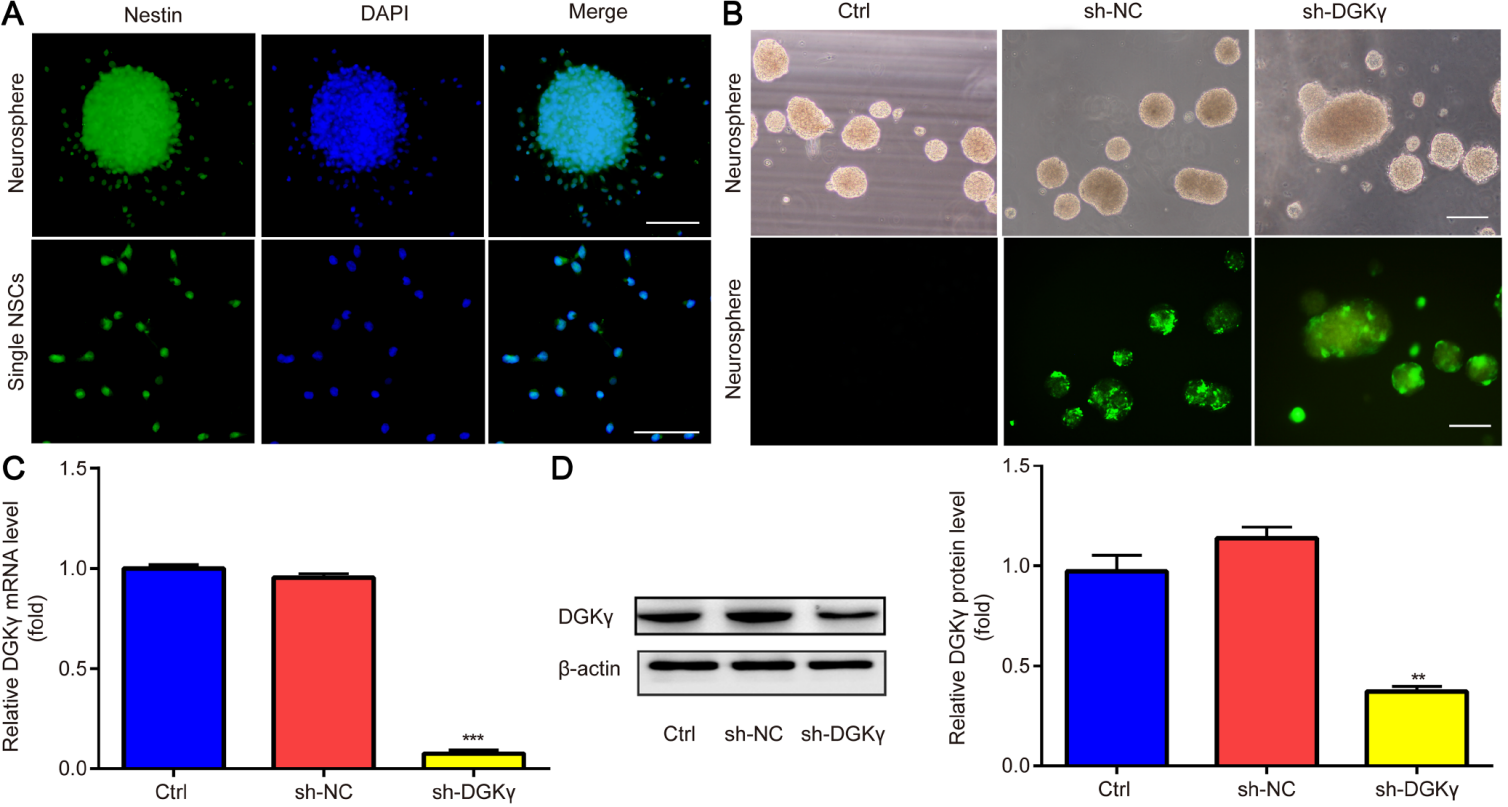
Figure 4 Identification of NSCs and verification of *DGKγ* knockdown in NSCs (A) Neurospheres with outwardly migrating cells and single NSCs were identified via immunofluorescence staining for Nestin. (B) The states of neurospheres infected with sh-DGKγ are presented (top), and the corresponding shRNA delivery efficiency is shown as indicated by GFP fluorescence (bottom). (C and D) Downregulated mRNA and protein expressions of DGKγ were detected in the sh-DGKγ group by qRT‒PCR and western blot analysis, respectively. ***P < 0.001 vs sh-NC. Scale bar: 100 μm. n = 3.

For the identification of neurospheres and single NSCs, we carried out immunofluorescence staining for Nestin. Most of the cells (> 90%) were Nestin positive, and the attached neurospheres were surrounded by outwardly migrating cells. Moreover, single NSCs were identified by immunofluorescence staining for Nestin ( Figure 4A).

### Verification of *DGKγ* knockdown in NSCs *in vitro*

The efficacy of sh-DGKγ in knocking down endogenous DGKγ expression was subsequently evaluated. After 72 h of lentivirus infection, the states of the neurospheres are presented ( Figure 4B, top), and the shRNA delivery efficiency is shown as indicated by GFP fluorescence ( Figure 4B, bottom). qRT-PCR and western blot analysis revealed that DGKγ mRNA and protein levels were significantly lower in the sh-DGKγ group than in the sh-NC group ( Figure 4C,D), indicating that sh-DGKγ successfully knocked down endogenous DGKγ expression in NSCs.

### Downregulation of DGKγ inhibits NSC proliferation

**Figure 5:**
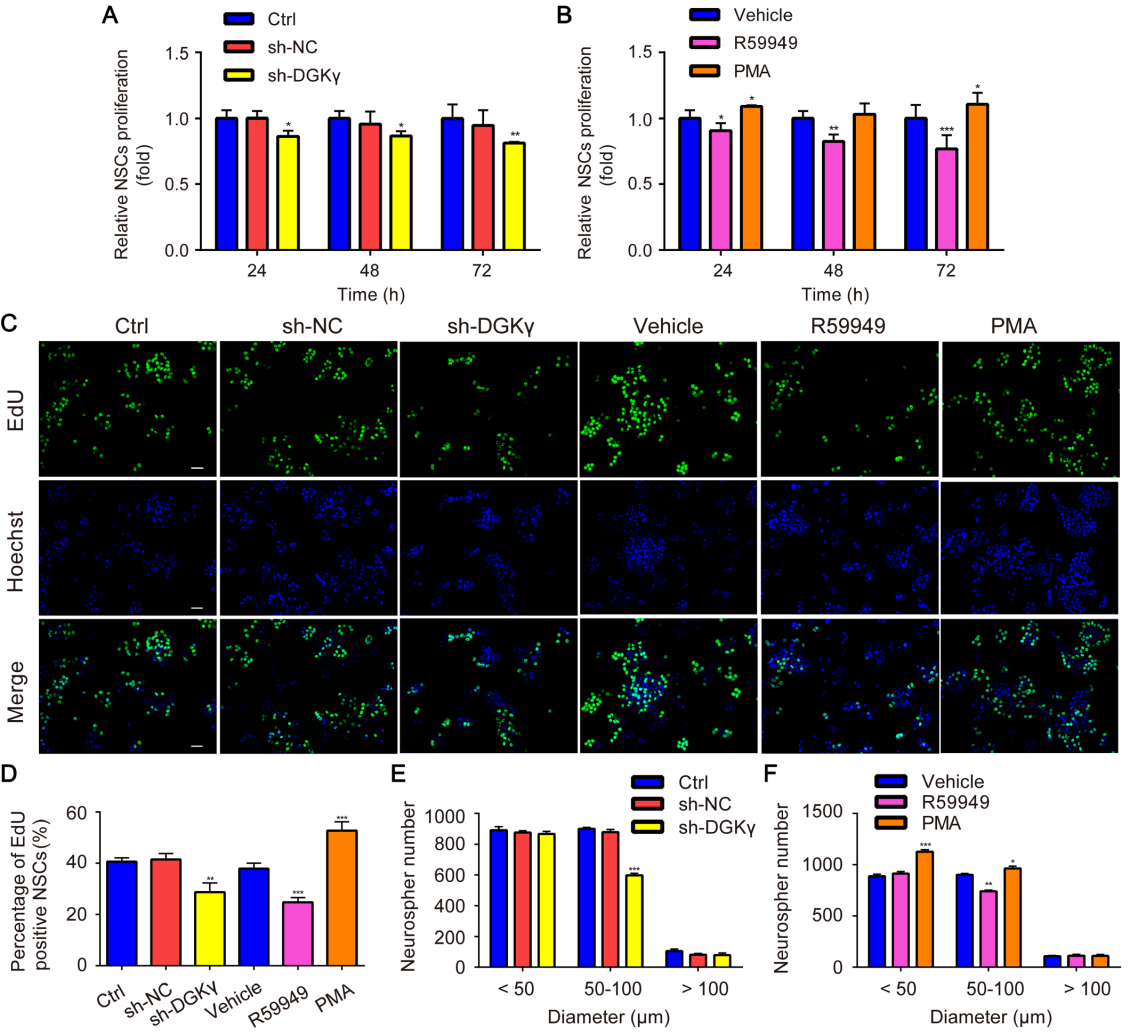
Figure 5 NSC proliferation was detected via a CCK-8 assay, an EdU assay, and a neurosphere formation assay (A,B) CCK-8 assays revealed that, compared with that of sh-NC-treated NSCs, NSC proliferation was significantly repressed by sh-DGKγ. Compared with that of vehicle-treated NSCs, NSC proliferation was markedly suppressed by R59949 and significantly promoted by PMA. (C,D) EdU assay revealed that, compared with those of sh-NC-treated cells, the number of EdU-positive cells was significantly lower in sh-DGKγ-treated cells than in vehicle-treated cells, and the number of EdU-positive cells was markedly lower in R59949-treated cells than in sh-NC-treated cells and significantly greater in PMA-treated cells. (E,F) Neurosphere formation assays revealed that, compared with those of sh-NC-treated neurospheres, medium-sized neurospheres (50‒100 μm) were markedly decreased by sh-DGKγ. Compared with those of vehicle-treated neurospheres, medium-sized neurospheres (50‒100 μm) were markedly decreased by R59949, whereas small-sized neurospheres (< 50 μm) and medium-sized neurospheres (50‒100 μm) were significantly increased by PMA. *P < 0.05, **P < 0.01, ***P < 0.001 vs sh-NC or vehicle. Scale bar: 100 μm. n = 4.

The proliferative activity of NSCs was analyzed via CCK-8 assay, EdU assay, and neurosphere formation assay. The results of CCK-8 assay revealed that, compared with that of the sh-NC group, the proliferation of NSCs was significantly inhibited by sh-DGKγ ( Figure 5A); additionally, compared with that of the vehicle group, the proliferation of NSCs was significantly suppressed by R59949 but significantly promoted by PMA ( Figure 5B).

Compared with those in sh-NC-treated NSCs, the number of EdU-positive NSCs in sh-DGKγ-treated NSCs was significantly lower. Additionally, compared with those in vehicle-treated NSCs, the number of EdU-positive NSCs in R59949-treated NSCs was significantly lower, but the number of EdU-positive NSCs in PMA-treated NSCs was significantly greater ( Figure 5C,D).

Thereafter, the ability of NSCs to create new neurospheres was evaluated. In the ctrl, sh-NC, and vehicle groups, only a few neurospheres were large in size and had a diameter greater than 100 μm. Most neurospheres had a diameter less than 100 μm, *i.e*., approximately half of the neurospheres were small in size (< 50 μm), and approximately half of the neurospheres were medium in size (50–100 μm). However, in the sh-DGKγ group, the number of medium-sized neurospheres but not small- or large-sized neurospheres markedly decreased compared with that in the sh-NC group ( Figure 5E). In addition, compared with that in the vehicle group, the number of medium-sized neurospheres markedly decreased in the R59949 group, whereas the number of small- and medium-sized neurospheres significantly increased in the PMA group ( Figure 5F). These results suggest that R59949 restrains the growth of small neurospheres into medium-sized neurospheres, thus slowing the proliferation of NSCs; however, PMA induces the growth of single NSCs into small neurospheres, as well as the growth of small neurospheres into medium-sized neurospheres, thus promoting the proliferation of NSCs.

### Downregulation of DGKγ inhibits NSC migration

**Figure 6:**
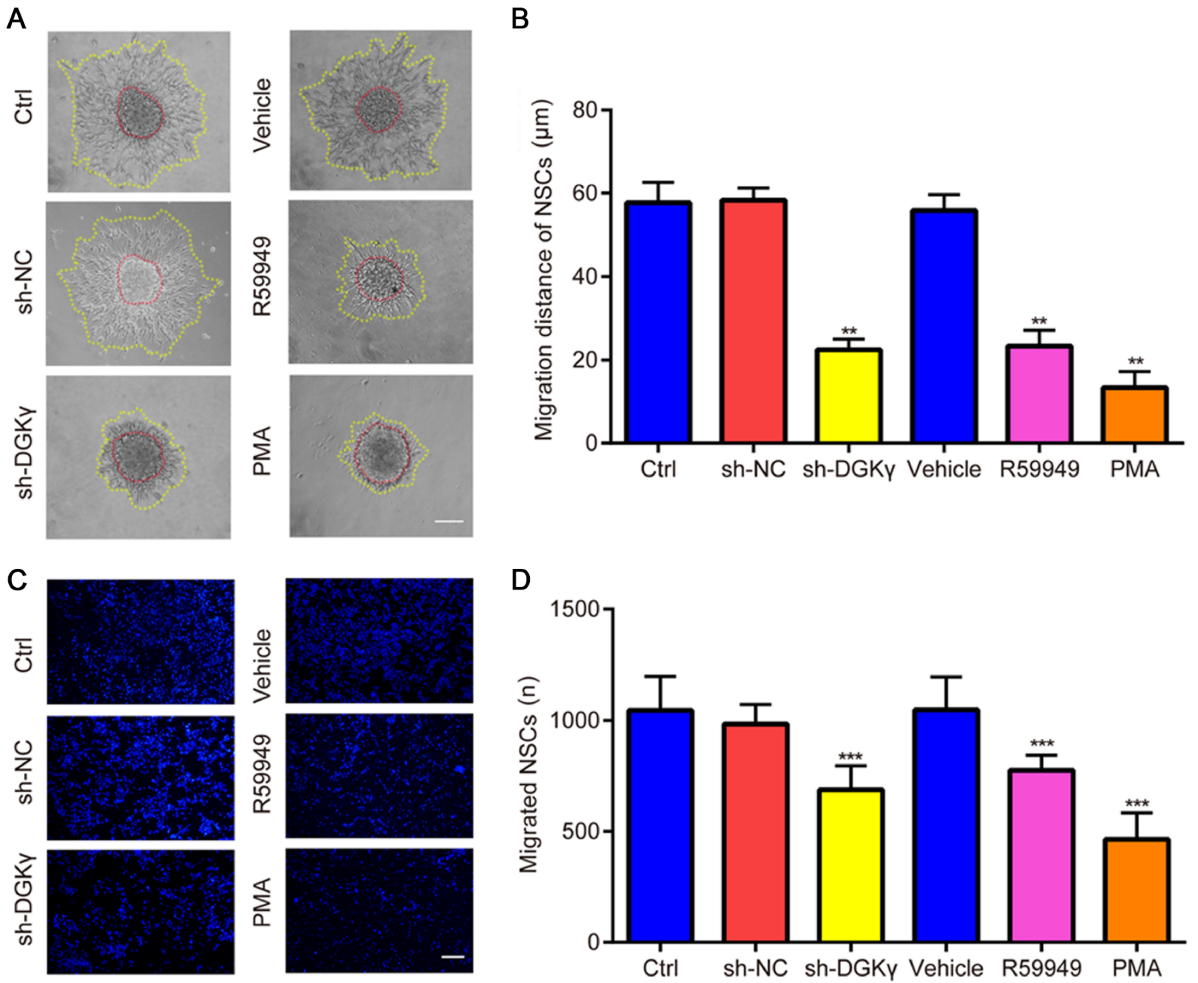
Figure 6 Detection of NSC migration via neurosphere migration and Transwell chamber assays (A,B) Neurosphere migration assays revealed that, in contrast to that of sh-NC-treated NSCs, the migration distance of NSCs was significantly decreased by sh-DGKγ; similarly, in contrast to that of vehicle-treated NSCs, the migration distance of NSCs was significantly decreased by R59949 and PMA. (C) Vertical cell migration was tested by a transwell chamber assay, and fluorescence images of the nuclei of transmigrated NSCs stained with DAPI are presented. (D) The number of transmigrated NSCs was calculated, and the results revealed that, compared with those in the sh-NC group, the number of cells that migrated to the lower surface of the transwell was lower in the sh-DGKγ group; likewise, compared with those in the vehicle group, the number of cells that migrated to the lower surface of the transwell was lower in the R59949 and PMA groups. **P < 0.01, ***P < 0.001 vs sh-NC or vehicle. Scale bar: 50 μm. n = 3.

NSC migration was subsequently evaluated via neurosphere migration assay and transwell chamber assay. The results of the neurosphere migration assay revealed that the sizes of the plated neurospheres varied; most of them were small (< 50 μm) or medium-sized (50–100 μm). The migration distance of NSCs in the sh-DGKγ group was significantly shorter than that in the sh-NC group; similarly, the migration distance of NSCs in the R59949 group and PMA group was significantly shorter than that in the vehicle group ( Figure 6A,B). Moreover, Transwell chamber assay results revealed that, compared with those in the sh-NC group, fewer NSCs migrated to the lower surface of the transwell in the sh-DGKγ group; likewise, compared with those in the vehicle group, fewer NSCs migrated to the lower surface of the transwell in the R59949 and PMA groups ( Figure 6C,D). These data indicated that downregulation of DGKγ inhibits NSC proliferation and migration.

### Downregulation of DGKγ increases the DAG content and p-PKCδ level in NSCs

**Figure 7:**
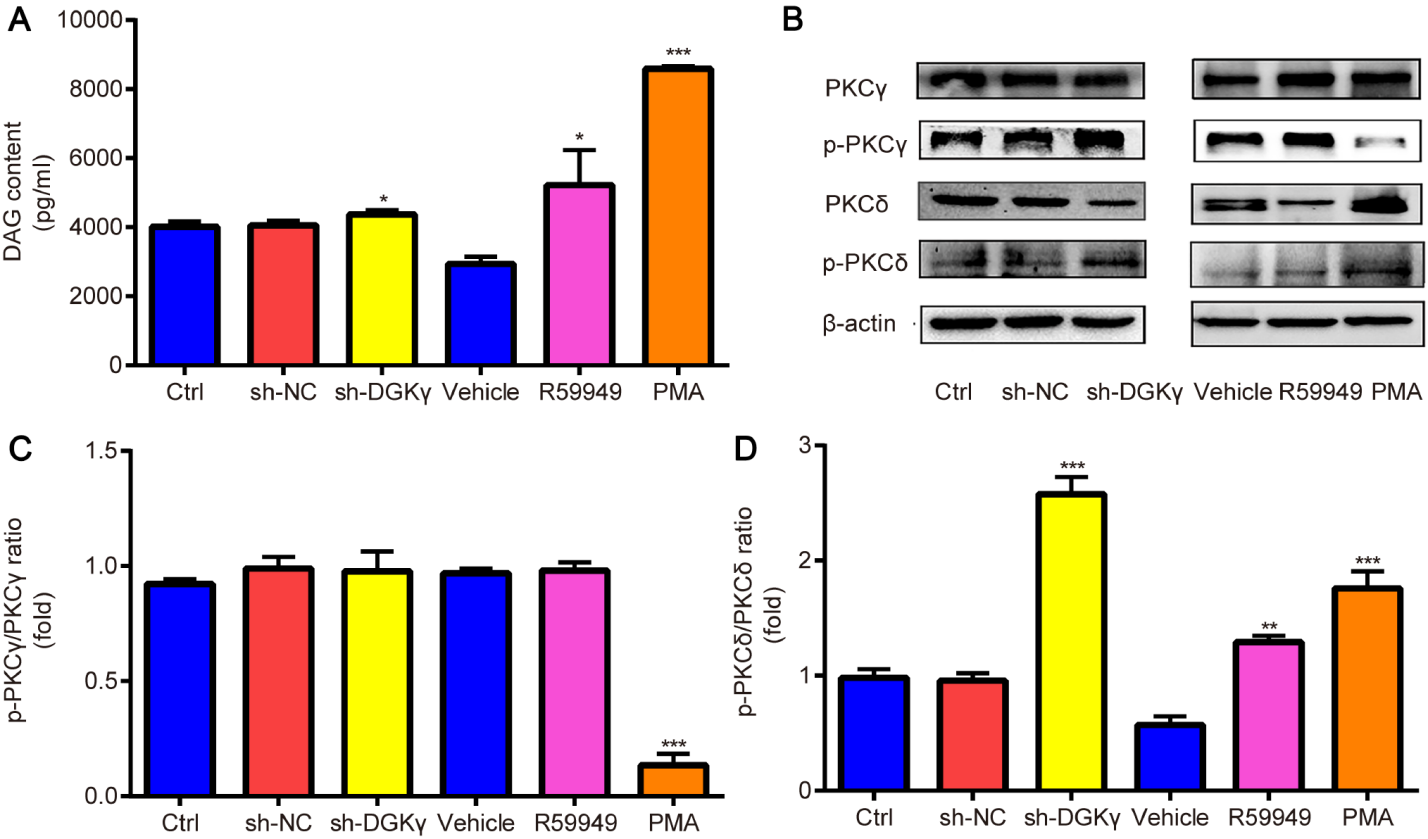
Figure 7 Changes in DAG content and p-PKC subtypes during NSC migration (A) DAG contents were determined by ELISA, which revealed that the DAG contents were significantly increased in sh-DGKγ-treated cells compared with those in the sh-NC-treated cells and significantly increased in R59949- and PMA-treated cells compared with those in the vehicle-treated cells. (B) Representative western blots of p-PKCγ and p-PKCδ in NSCs were quantified via western blotting. (C,D) Densitometric analysis of the western blots revealed that the p-PKCγ/PKCγ ratio was significantly lower in the PMA group than in the vehicle group; however, the p-PKCδ/PKCδ ratio was significantly greater in the sh-DGKγ group than in the sh-NC group, which was also significantly greater in the R59949 and PMA groups than in the vehicle group. *P < 0.05, **P < 0.01, ***P < 0.001 vs sh-NC or vehicle. n = 3.

Changes in the DAG content were detected via ELISA, which revealed that the DAG content was significantly greater in sh-DGKγ-treated cells than in sh-NC-treated cells and was significantly greater in R59949- and PMA-treated cells than in vehicle-treated cells ( Figure 7A). Changes in p-PKCγ, PKCγ, p-PKCδ, and PKCδ protein levels were detected via western blot analysis, which revealed that the p-PKCγ/PKCγ ratio was significantly lower in the PMA group than in the vehicle group ( Figure 7B,C); however, the p-PKCδ/PKCδ ratio was significantly greater in the sh-DGKγ group than in the sh-NC group and was significantly greater in the R59949 and PMA groups than in the vehicle group ( Figure 7B,D). These data indicated that downregulation of DGKγ increases the DAG content and p-PKCδ level in NSCs.

## Discussion

Herein, the effects and possible mechanisms of DGKγ on NT development and NSC proliferation and migration were investigated both *in vivo* and *in vitro*. The findings revealed that DGKγ, which is expressed in the developing NT, was coexpressed with p-PKCγ and p-PKCδ in NCSs; additionally, the knockdown of *DGKγ* suppressed NSC proliferation and migration. Taken together, the results of the present study demonstrated that DGKγ promoted NSC migration via the DAG/PKCδ pathway.

On the basis of the atlas of embryonic development in rats and mice [ 21, 22], a crucial period for NT formation is estimated to be E10‒E11.5 in rat embryos. In the present study, WMISH revealed that DGKγ mRNA was expressed primarily along the NT line at E10 and E11; moreover, immunohistochemistry revealed that, compared with the ctrl line at E11, NSCs were expressed, and the DGKγ protein was expressed in the neuroepithelium cells of the embryos from E10 to E11.5. Collectively, these results demonstrated the involvement of DGKγ in NT development through the regulation of the function of NSCs.

The proliferation and migration of NSCs are prerequisites for NT development; consequently, the effects of DGKγ on NSC proliferation and migration were subsequently explored. In the present study, sh-DGKγ was shown to inhibit NSC proliferation. To further verify the involvement of DGKγ in NSC proliferation, NSCs were treated with R59949 and PMA. In the DAG/PKC signaling pathway, reduced DGKγ attenuates DAG degradation, which activates PKC to promote cell proliferation [26]. R59949, an inhibitor of DGK that increases DAG and activates PKC, has a strong inhibitory effect on DGKγ by binding to the activity center [27]. PMA is a DAG analog that induces PKC activation [28]. It is thus reasonable to expect that R59949 and PMA induce similar effects. However, the current study revealed that R59949 inhibited, while PMA accelerated, NSC proliferation. These discrepancies may be attributed to the occurrence of phenomena that are not explained by the simplified effects of the two chemicals. One possible reason is the ability of PMA to activate various PKC species irreversibly. One possible reason is that there is a mechanism mediated by PKC (but independent of DGKγ), which induces the proliferation of NSCs. Another possible reason is that the mechanism is dependent on DGKγ but independent of PKC, which favors cell proliferation. Another possible reason is that the activation of DGKγ can regulate other signaling pathways, such as the mTOR pathway, which is involved in proliferation [ 29, 30]. In summary, the effect of DGKγ on NSC proliferation was speculated to be independent of the DAG/PKC signaling pathway. DGKγ can be transported from the cytoplasm to the nucleus under serum-starved conditions. DGKγ-negative cells exhibit decreased proliferative ability, whereas the nuclear translocation of DGKγ is independent of its kinase activity [ 31, 32]. Moreover, the current study revealed that DGKγ was expressed in the cytoplasm and nucleus; therefore, DGKγ in the nucleus might regulate NSC proliferation.

DGKγ has been reported to function as a tumor suppressor by inhibiting cell migration [ 33, 34]. In contrast, the present study demonstrated that the knockdown of *DGKγ* inhibited NSC migration. In addition, PKC activation and DGKγ blockade caused similar inhibitory effects, indicating that DGKγ regulates NSC migration via its kinase activity, which may be attributed to the DAG/PKC signaling pathway.

Among all the PKC subtypes, PKCγ and PKCδ have been shown to interact with DGKγ. For the interaction between PKCγ and DGKγ, mass spectrometric analysis and mutation studies revealed that PKCγ activates DGKγ by phosphorylating Ser-776 and Ser-779 at its accessory domain [35]. Additionally, PKCγ activity is increased in the cerebellum of *DGKγ*-KO mice, and DGKγ directly interacts with PKCγ [17], demonstrating spatiotemporal regulation between PKCγ and DGKγ, which is precisely controlled by their direct interaction but not the classic DAG/PKC signaling pathway. With respect to the interaction between PKCδ and DGKγ, extracellular signals can induce the translocation of DGKγ from the cytoplasm to the cell membrane, resulting in its colocalization with PKCδ [16]; moreover, the phosphorylation of PKCδ is necessary for vascular smooth muscle cell migration [36]. Therefore, the protein expression levels of p-PKCγ and p-PKCδ were detected via immunofluorescence double staining in the present study. p-PKCγ and p-PKCδ were found to be expressed in the cytoplasm and nucleus of neuroepithelial cells, and each of these proteins was colocalized with DGKγ. Moreover, p-PKCγ, PKCγ, p-PKCδ, and PKCδ protein levels, as well as the DAG content, were detected in cultured NSCs via western blot analysis and ELISA. The p-PKCδ/PKCδ ratio was significantly increased, whereas the p-PKCγ/PKCγ ratio was significantly decreased by PMA, demonstrating that downregulation of DGKγ (blocking DGK) and activation of PKC significantly increased p-PKCδ protein level, which is consistent with the significant increase in DAG content. Therefore, PKCδ, but not PKCγ, is significantly activated by PMA or increased DAG content in NSCs. Taken together, these findings indicate that the DAG/PKCδ signaling pathway accelerates DGKγ-mediated NSC migration.

In addition, on the one hand, knockdown of *DGKγ* was found to cause a minor increase in the DAG content, indicating that other endogenous DKGs, such as DGKζ, might be active. On the other hand, PMA significantly increased the DAG content, suggesting that some PKC subtypes increase the DAG content via a feed-forward mechanism, as activated PKCα inhibits the activity of DGKζ via the phosphorylation of DGKζ [37].

**Figure 8:**
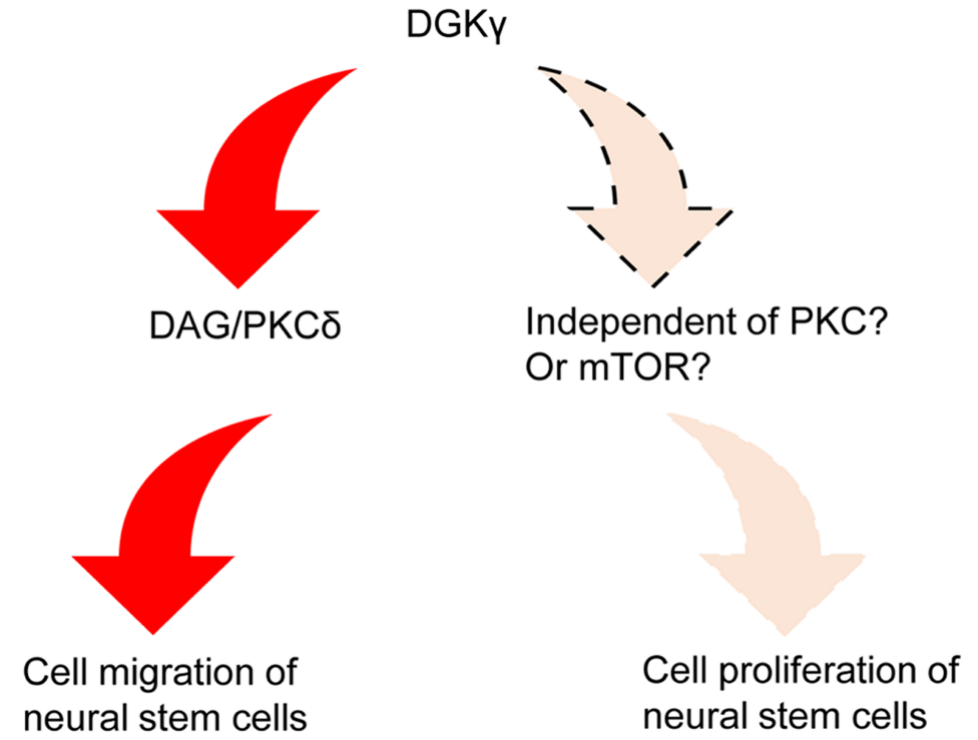
Figure 8 Schematic diagram of the role of DGKγ in NSC migration and proliferation A schematic diagram showing that the DAG/PKCδ signaling pathway is involved in NSC migration and that the mTOR signaling pathway, independent of PKC, may be involved in NSC proliferation.

In conclusion, the mRNA and protein expression levels of DGKγ in NTs indicate that DGKγ is associated with NT development. Downregulating DGKγ expression and blocking DGK activity inhibited NSC proliferation and migration *in vitro*. As shown in Figure 8, DGKγ regulates NSC migration via the DAG/PKCδ signaling pathway, whereas DGKγ regulates NSC proliferation via other potential signaling pathways ( *e*. *g*., the mTOR signaling pathway or signaling pathways that are independent of PKC). Nevertheless, there are some limitations in the present study, i.e., (1) No *in vivo* experiment was done to verify whether the knockdown of *DGKγ* impairs the development of NT; (2) The molecular mechanism was not studied extensively; (3) Transwell experiments cannot mimic migration in developing NTs; (4) It is insufficient to define NSCs using Nestin only. These issues will be addressed and/or improved in our future research.
